# Toenail manganese as a potential biomarker for *in utero* and early childhood exposure studies

**DOI:** 10.1038/s41598-018-35112-0

**Published:** 2018-11-19

**Authors:** Shannon S. Cigan, Patricia M. McGovern, Kriti Choudhary, Neely C. Miller, Michael Georgieff, Raghavendra Rao, Irina Stepanov

**Affiliations:** 1 School of Public Health, Division of Environmental Health Sciences, University of Minnesota, Minneapolis, MN 55455 USA; 2 Medical School and Department of Pediatrics, Division of Neonatology, University of Minnesota, Minneapolis, MN 55454 USA; 3 Center for Neurobehavioral Development, University of Minnesota, Minneapolis, MN 55414 USA; 4 Masonic Cancer Center, University of Minnesota, Minneapolis, MN 55455 USA

## Abstract

Elevated *in utero* and early childhood exposure to manganese may have adverse effects on neurodevelopment. We conducted preliminary analyses to evaluate toenails as a matrix for investigating manganese exposure in infants. Infant and maternal toenail and hair samples were collected from 25 infants (7 months old) and their mothers. A subset of mothers was recruited in the third trimester of pregnancy and some also provided pre-natal toenail, hair, and blood samples, cord blood, and additional post-natal samples. Collected samples were analyzed by inductively coupled plasma mass-spectrometry. Toenail manganese levels in infants ranged from below the limit of detection (LOD) to 2.80 µg/g. Only 1 toenail sample and 4 hair samples contained levels of manganese below LOD. Associations between infant and maternal biomarkers were not statistically significant. Analysis of multiple post-natal toenail samples from a single infant-mother pair showed an increase in the infant’s toenail manganese and a decrease in maternal toenail manganese over the first year of the infant’s life. Overall, our findings suggest that toenails may serve as a valuable biological matrix for measuring manganese exposure in newborns and infants; however, additional studies are needed to determine the impact of the timing of toenail sample collection on its utility in assessing early life exposure and health outcomes.

## Introduction

Manganese (Mn) is an essential nutrient that plays an important role in normal growth and development, including a variety of physiological processes^1–4^; however, emerging literature suggests that childhood exposure to even low levels of environmental Mn may have adverse neurodevelopment effects and could be a contributor to the rise in neurobehavioral disorders^5–10^. For instance, studies carried out in Canada and Bangladesh show that higher levels of Mn in drinking water were associated with hyperactive and oppositional behaviors^11^, lower IQ scores^6^, and reduced mathematics achievement scores^12^. Other studies employed a biomarker-based approach and reported that high Mn levels in children’s blood and hair are associated with poor memory^13–15^, low cognitive scores^16,17^, and impaired motor function^15^. Despite this growing evidence, U.S.-based studies of Mn exposure and neurobehavioral outcomes in children are scarce: one exploratory study demonstrated an association between Mn levels in tooth enamel and behavioral disinhibition in children^18^, and another recent study found an association between high levels of Mn in blood and hair with lower IQ scores in children living near a ferromanganese refinery in Ohio^19^.

Most of the studies investigating Mn exposure have analyzed its effects in school-aged children; however, *in utero* and early childhood exposures are of particular concern because this is a critical time in brain development. Furthermore, while Mn levels are closely regulated by homeostatic mechanisms in adults, these mechanisms may be inefficient in newborns and infants resulting in increased absorption efficiency and reduced biliary excretion^20,21^. Formula-fed infants may be particularly vulnerable if the formula is reconstituted with Mn-contaminated water. Therefore, there is an urgent need for studies to better understand the potential link between early life Mn exposures and subsequent adverse neurobehavioral outcomes.

Biomarkers used in most of the previous studies may not be suitable for testing in newborns or infants. Blood sample collection is invasive and has high variability and a short half-life (13–74 days), factors which may explain the findings of weak or nonexistent associations between this biomarker and environmental measures of exposure to Mn^22–24^. Levels of Mn in cord blood are 2-fold higher than in maternal blood^25,26^, and the association of Mn levels in blood with neurodevelopmental outcomes in children is inconsistent^5^. Hair is very porous and can be externally contaminated with Mn and, therefore, may not be representative of internal dose^27^. In addition, Mn levels in hair can be influenced by Mn-contaminated water for washing, hair treatment, hair pigmentation, growth rate and varying sample cleaning procedures^28^. Lastly, hair may not always be readily available in infants, due to their varying hair growth. Both blood and hair reflect only relatively short-term exposures. Analysis of deciduous teeth can provide important retrospective information about in utero and early childhood exposures; however, it takes years for the teeth to shed which prevents their use in studies of newborns and infants focused on early life neurobehavioral outcomes.

Toenail Mn measurements could potentially serve as better biomarkers for the monitoring of Mn exposure in infants and *in utero*. The human toenail has a very slow growth rate (1.62 mm/month)^29^ and, therefore, biomarker levels in toenail clippings represent cumulative exposures over 6–12 months. Toenails start forming in embryos at 9–10 weeks old, suggesting that toenail Mn could be used to assess *in utero* cumulative exposure to Mn. Unlike hair, toenails are always present in relatively similar amounts in newborns and infants. In addition, external contamination of toenails with Mn is less likely to occur than that of hair, and any trace contamination can be easily washed away with little to no effect on the toenail elemental content^29^. A single recent study, conducted in Bangladesh, used toenails to investigate prenatal exposure to Mn from drinking water in mother-infant pairs; however, the findings of the correlation between maternal and infant toenail Mn levels were inconsistent across the two regions included in the study^30^.

We conducted a pilot study to evaluate the potential utility of levels of Mn in toenails as a biomarker of early life exposure to this element. Toenail samples were collected from infants and their mothers, and analyses of hair and blood were also carried out to assess the relationships of Mn levels among various matrices.

## Results

**Figure 1:**
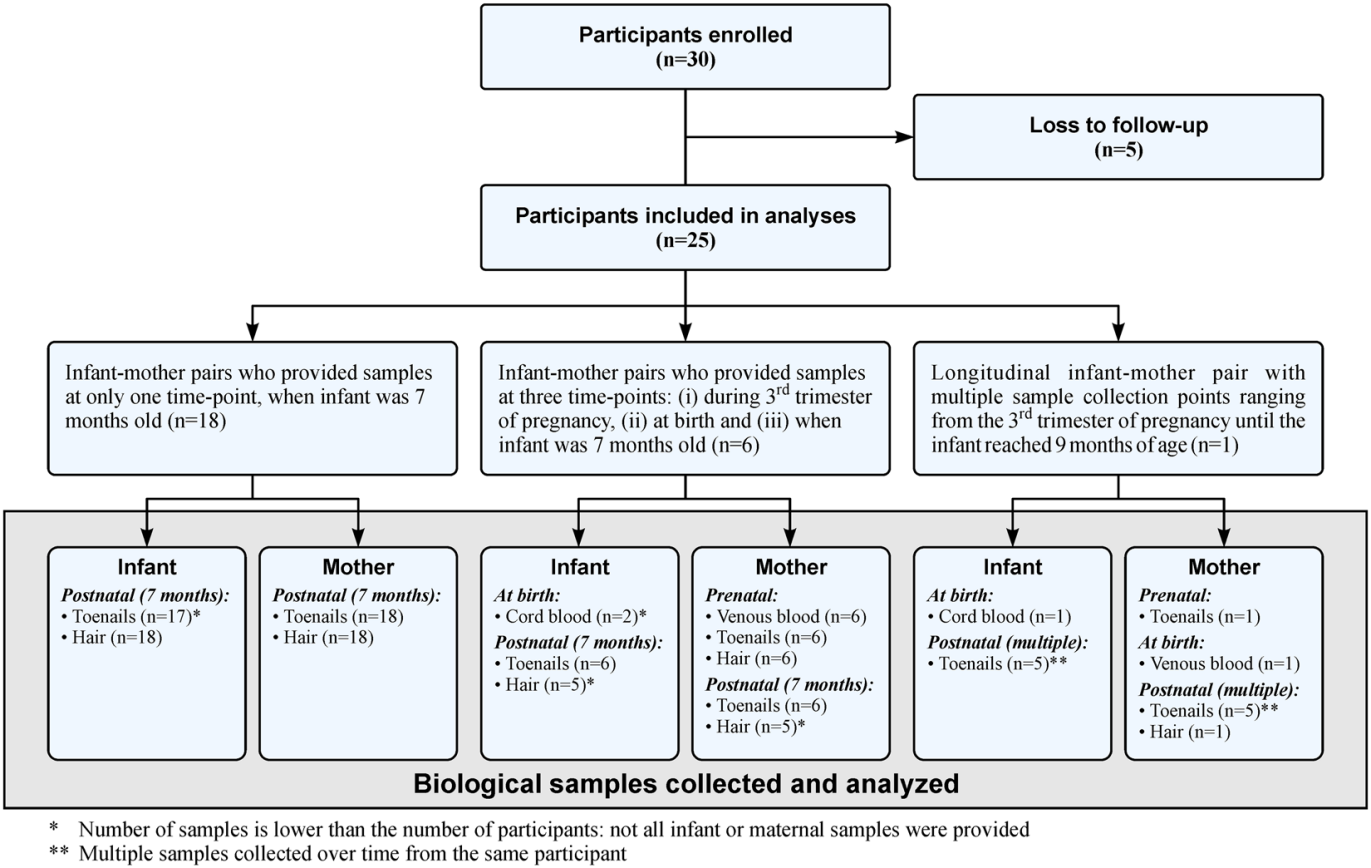
Participant enrollment and sample collection.

A total of 25 infant-mother pairs were recruited for this pilot study (Fig. 1). Of these, 6 mothers were part of a subset that was recruited early in pregnancy, and they provided samples in the third trimester of pregnancy and at the time of birth. From the longitudinal infant-mother pair, the mother provided toenail samples during her third trimester of pregnancy, maternal venous blood and cord blood at delivery, one maternal hair sample, and infant and maternal toenail samples over an 11-month time period after birth.

**Figure 2:**
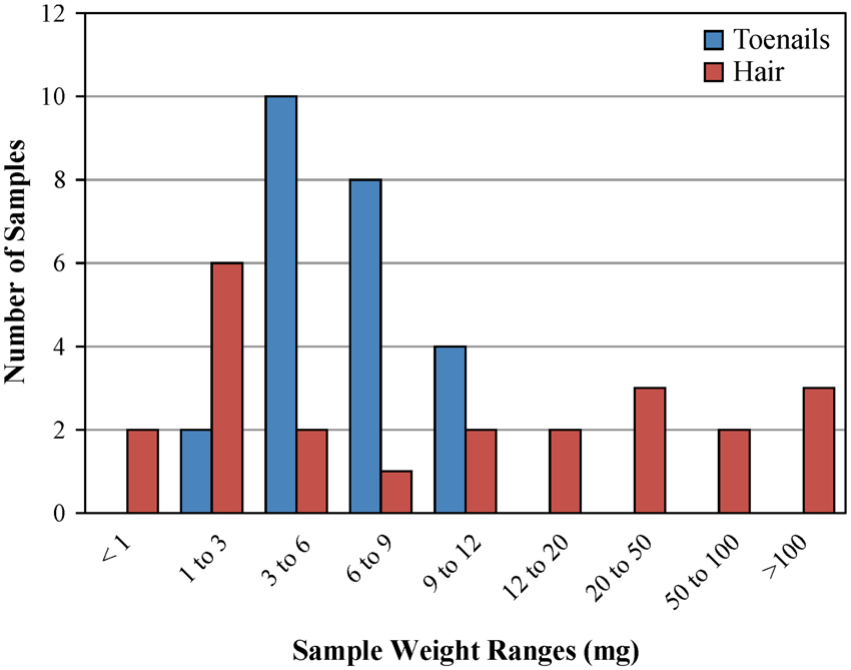
Distribution of infant toenail and hair sample weights.

Manganese levels in infant and maternal samples collected when the infants were 7 months old are shown in Table 1. The distribution of sample weights for infant toenails and hair are presented in Fig. 2. Overall, toenails contained lower Mn levels than hair, and Mn concentrations in infants were lower than those in the mothers. Toenail and hair samples collected from infants showed substantial variation in Mn concentrations, ranging from below the limit of detection (LOD) to 4.59 µg/g and from below LOD to 6.08 µg/g, respectively. Only one toenail sample and 4 hair samples contained levels of Mn below LOD. Correlation between infant Mn concentrations in toenails and hair was not statistically significant (R = 0.16, *p* = 0.47; Table 2). In mothers, both toenail and hair Mn levels were higher than in infants, ranging from 0.19 to 20.98 µg/g and 0.09 to 13.35 µg/g, respectively. The correlation between Mn concentrations in maternal toenails and hair did not reach statistical significance (R = 0.34; *p* = 0.11). Similarly, statistical significance was not reached for the correlations between Mn levels in infant and maternal toenails (R = 0.30; *p* = 0.16) or between infant and maternal hair (R = 0.34; *p* = 0.12) (Table 2).

**Table 1:** Levels of Mn in samples collected from 7-month-old infants and their mothers^a^.

Sample type		Infants	Mothers
Toenails (µg/g)	N (samples) Mean (SD) Range, min–max	23 0.73 (1.12) <LOD^b^ − 4.59	24 1.96 (4.15) 0.19–20.98
Hair (µg/g)	N (samples) Mean (SD) Range, min–max	23 0.94 (1.40) <LOD^b^ − 6.08	23 2.90 (3.76) 0.09–13.35

^a^Data excluding longitudinal infant-mother pair, ^b^<LOD, Mn below the limit of detection.

**Table 2:** Relationship among biomarkers in samples from 7-month-old infants and their mothers (23 infant-mother pairs)^a,b^.

	Infant Hair	Maternal Toenail	Maternal Hair
Infant Toenail	0.16 (*p* = 0.47)	0.30 (*p* = 0.16)	0.10 (*p* = 0.66)
Infant Hair		−0.12 (*p* = 0.57)	0.34 (*p* = 0.12)
Maternal Toenail			0.34 (*p* = 0.11)

^a^Data are log-transformed to establish normal distribution.

^b^Data excluding longitudinal infant-mother pair. Each cell contains Pearson correlation coefficients R and p-value.

Levels of Mn in tap water ranged from <10 µg/L to 290 µg/L. Analysis of the relationship of biomarkers with tap water Mn levels revealed no correlation with either infant or maternal toenail Mn levels (R = 0.22; *p* = 0.41 or R = 0.29; *p* = 0.28, respectively). There was no correlation with Mn levels in the tap water source and infant hair (R = 0.31; *p* = 0.26); however, maternal hair (R = 0.74; *p* = 0.01) significantly correlated with Mn levels in the tap water source.

**Figure 3:**
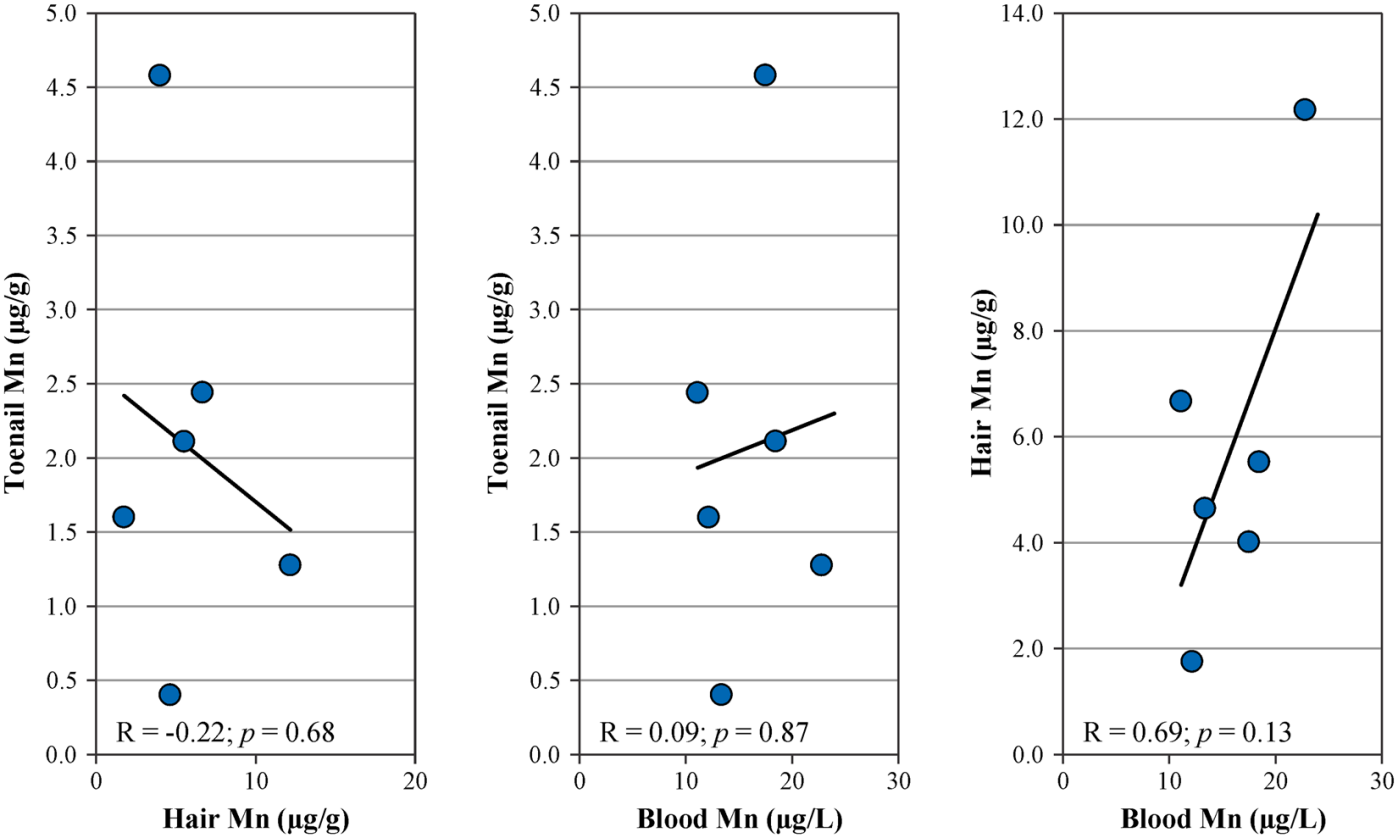
Relationship among Mn biomarkers in subset of mothers who provided samples in the third trimester of pregnancy (N = 6).

In the subset of samples collected from 6 mothers, who provided prenatal and postnatal samples, Mn levels in prenatal samples were generally higher than in the postnatal samples (Table 3): mean toenail Mn levels decreased from 2.07 µg/g in prenatal to 1.43 µg/g in postnatal toenails (*p* = 0.22) and mean hair Mn levels decreased from 5.80 µg/g in prenatal to 5.15 µg/g in postnatal samples (*p* = 0.05). The relationship among prenatal Mn biomarkers in this subgroup is shown in Fig. 3. Toenail Mn concentrations did not correlate with either hair (R = −0.22; *p* = 0.68) or blood (R = 0.09; *p* = 0.87). The correlation between maternal hair and blood did not reach statistical significance (R = 0.69; *p* = 0.13).

**Table 3:** Levels of Mn in maternal samples and cord blood from six participants with available prenatal and postnatal samples^a^.

Sample type		Time of collection			p-value^b^
		Pregnancy (third trimester)	Delivery	7 months (post-natal)	
Toenails (µg/g)	N (samples) Mean (SD) Range, min–max	6 2.07 (1.42) 0.40–4.58		6 1.43 (1.13) 0.55–3.32	0.22
Hair (µg/g)	N (samples) Mean (SD) Range, min–max	6 5.80 (3.53) 1.76–12.17		5 5.15 (3.53) 1.55–10.46	0.05
Blood (µg/L)^c^	N (samples) Mean (SD) Range, min–max	6 15.89 (4.46) 11.12–22.76	2^d^ 49.40 (13.92) 39.56–59.24		

^a^Data excluding longitudinal infant-mother pair, ^b^p-values for pairwise T-test for pre- and post-natal measures, ^c^venous blood for pregnant mothers and cord blood for infants, ^d^not all samples were provided.

**Figure 4:**
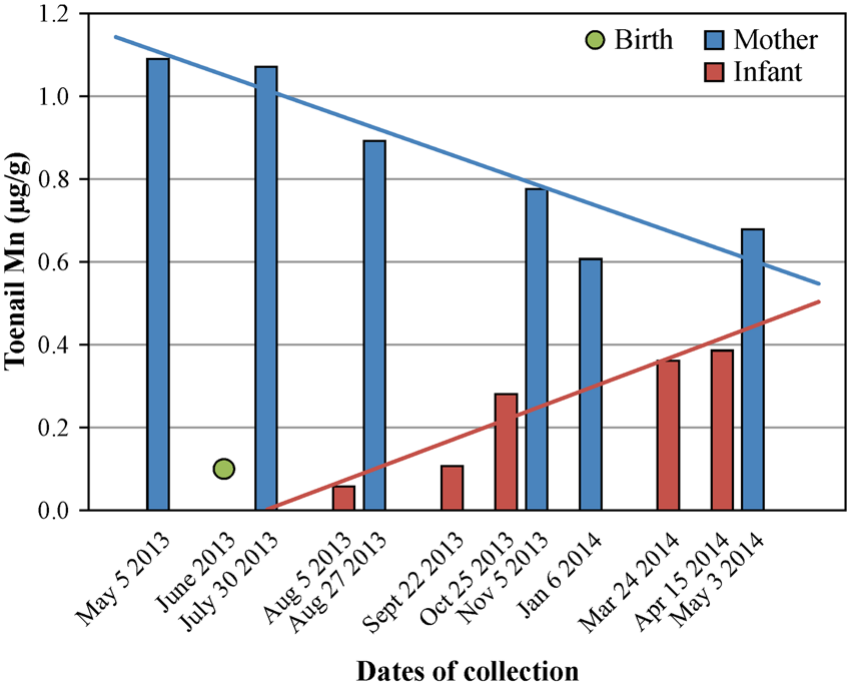
Levels of Mn in toenails from the single longitudinal infant-mother pair.

In the single longitudinal infant-mother sample set, toenail Mn in the infant increased from 0.06 to 0.39 µg/g and the mother’s toenail Mn decreased from 1.09 to 0.68 µg/g over a 12-month period of time (Fig. 4). The infant’s cord blood Mn level was 30.92 µg/L, and the maternal venous blood Mn level was 17.49 µg/L.

## Discussion

While fetal and early childhood development may be particularly susceptible periods to the adverse neurobehavioral effects of elevated Mn exposures, the best approaches to measuring such exposures are not known. Given the limitations of hair, blood, and deciduous teeth as the biological matrices for the measurement of Mn exposures in newborns and infants, we aimed to assess toenails as an alternative biomarker. Our primary goals in this study were to (i) test the feasibility of Mn measurement in the toenail samples collected from infants, (ii) evaluate the range of interindividual variation of the toenail Mn levels in infants, and (iii) analyze the relationships of infant toenail Mn levels with those in their hair as the most commonly used biomarker in the previous studies of childhood Mn exposure.

The results of our study support the feasibility of infant toenail collection and analysis. We did not encounter resistance to toenail collection: of the 25 recruited mothers, only one did not provide her infant’s toenail sample. Infant toenail sample weights ranged from 2.7 to 11.3 mg, with 75% of samples being in the relatively narrow range between 3 and 9 mg (Fig. 2). For the infants’ hair, the weights of many samples were much higher than the toenail range, up to 271.3 mg; however, there was much larger weight variation across samples, with approximately 35% having less than 3 mg of hair. Furthermore, despite the generally lower levels of Mn in toenails as compared to hair, only one infant toenail sample had Mn levels below LOD. At the same time, Mn levels in 4 infant hair samples were below LOD, most likely due to the insufficient mass of sample (3 of these samples had less than 2 mg hair). Also, not all mothers provided cord blood, which was attributed to the sample collection difficulties using a vacutainer and the overall stressful environment during delivery. This outcome again supports the need for robust biomarkers to assess *in utero* exposure to trace elements. Lastly, in the single longitudinal infant-mother pair, the mother collected toenails 5 times over the first year of her child’s life, then sent them in a single shipment to the laboratory. This approach can be used in future studies to monitor the trends in the infant Mn biomarkers, while minimizing the burden and the expense of sample collection and shipment. Taken together, our observations support our hypothesis that toenails represent a more robust matrix for Mn exposure measurements, compared to hair or cord blood, in infants and newborns.

Toenail Mn levels measured in infants in our study were lower than those reported for infants in Bangladesh^30^. This could potentially be due to the differences in the levels of environmental and dietary Mn exposure. For instance, the levels of Mn in drinking water and infant hair were both higher in the Bangladesh study, compared to our data. In addition, toenail Mn levels in infants in our study varied substantially (Table 1), which suggests the potential impact of diet and/or the environment, including maternal exposures, on the levels of infants’ Mn body burden. For example, some infants in our study were breastfed for various durations of time, but all mothers who breastfed augmented their breastfeeding with formula. While there was no association between the toenail Mn levels and the concentration of Mn in water, further analyses are underway to examine the more detailed and comprehensive collected information on the external measures of exposure in infants and mothers, such as drinking water consumption (including as part of baby formula, when applicable), dietary Mn intake, and other sources. Inter-individual differences in the rate of toenail growth could also be contributing to the variations in toenail Mn levels across infants; however, due to the slow nail growth such contribution is expected to be minimal.

The increase in toenail Mn levels after birth, as seen in the single infant with multiple toenail samples, is a potentially important finding (Fig. 4). The observed changes may be due to environmental exposures to Mn and/or due to changes in the body’s ability to properly regulate Mn levels. This finding suggests that there may be a dynamic period of time when toenail biomarker levels are continuously changing in infants and, therefore, should be avoided in designing research studies that employ toenail biomarkers for the assessment of environmental exposures. Our observation also suggests that in the first 4–6 months of life, while Mn homeostasis is being achieved, infants may be most vulnerable to excess Mn exposures. Although a toenail sample was not collected from this particular infant at 7 months of age, the trend line in Fig. 4 shows that the infant’s toenail Mn level at that time-point was approximately 0.3 µg/g. This is within the range of toenail Mn levels in the larger group of infants; however, some infants had much higher levels which could be a potential reason for concern (Table 1). Larger longitudinal studies are needed to determine whether the phenomenon of increasing toenail Mn levels after birth is common for all infants.

Infant hair Mn levels in our study were comparable to the previously published data on hair Mn levels in school-aged children^3,13,31^. The substantial interindividual variation in hair Mn levels among infants in our study is consistent with the toenail Mn variation and further supports the potential effect of differing Mn exposure among the infants. In evaluating the relationship between water Mn levels and hair Mn levels, we did not find a correlation with the tap water source provided (R = 0.31; *p* = 0.26). It is not clear whether an increase in the hair Mn levels occurs after birth, as was observed for toenails (Fig. 4), and infant hair samples are generally too small to look at for such time trends. The differences in the rate of longitudinal Mn level changes in infant toenails and hair over the first year of life could potentially be responsible for the lack of correlation between these matrices in our study (Table 2). Only a small number of women provided cord blood samples for our study, which is consistent with our expectation that cord blood may not be a robust matrix for the assessment of in utero exposures. The small number of samples precludes an in-depth analysis of the results for the cord blood; however, consistent with the previous reports, the measured Mn levels in these samples were relatively high, more than 3-fold higher than Mn concentrations in maternal venous blood (Table 3). This may be attributed to the active transport of Mn across the placenta, the rapid growth of the fetus, and/or lack of well-developed mechanisms *in utero* that control Mn homeostasis^1,18,26,28,30^.

Maternal biomarkers could potentially serve as proxies for infant exposure in future studies; however, there was only a slight but non-significant correlation between maternal and infant biomarkers in our study. These results are in contrast with the report of maternal and infant Mn analyses by Rodrigues *et al*.^30^ which could be due to the differences in infant age between the two studies. The study by Rodrigues *et al*. collected toenail samples within one month after birth, and therefore, Mn levels in the collected samples were representative of *in utero* cumulative exposures. In our study, toenail samples were collected when the infants were 7 months old, and Mn levels in these samples could have been affected by the postnatal longitudinal changes. Furthermore, analysis of maternal toenail samples from our infant-mother longitudinal pair shows that there was a steady decline in maternal toenail Mn levels over the first year post-delivery (Fig. 4). Consistent with this observation, Mn levels in prenatal toenail samples were generally higher than in postnatal toenails in the 6 mothers who provided additional samples during the third trimester of pregnancy (Table 3). This trend could be attributed to the reported increase in maternal blood Mn levels during pregnancy, followed by the return to normal levels after delivery^25,32^. Despite the decline, maternal toenail Mn at 7 months post-delivery was higher than infant toenail Mn level at the same time-point: approximately 2-fold difference for the single infant-mother longitudinal pair (Fig. 4) and approximately 2.6-fold difference for the larger group of infant-mother pairs (estimated average, Table 1). Taken together, our observations suggest that there may be opposite longitudinal trends in infant and maternal Mn biomarker levels during the first year of an infant’s life, potentially explaining the lack of correlation between these biomarkers in our study. Additional analyses in the subset of 6 pregnant mothers, who provided toenail, hair, and blood samples during the third trimester of pregnancy, showed that blood Mn levels did not correlate with toenail Mn levels (R = 0.09; p = 0.87), but they did have a positive albeit not statistically significant (R = 0.69; p = 0.13) correlation with Mn levels in hair (Fig. 3). These observations are consistent with the expectation that biomarker levels in blood and in hair segments proximal to the scalp reflect more recent exposures, while toenail biomarkers cover a wide window of cumulative exposure^29^.

The limitations of our study include the small sample size which limits interpretation and generality of the results. Additional research with a larger sample size of infant-mother pairs with a variation of Mn exposure and/or multiple samples collected over the first year of the infants’ lives are needed to validate our findings. Additionally, sex may play an important role in Mn metabolism; however, due to the nature of our small study we lack the ability to distinguish sex-specific differences. Therefore, future larger studies should account for potential sex-specific differences.

In summary, findings from our pilot study support the feasibility of using infant toenails as the biological matrix for the analysis of in utero and early childhood exposures to Mn, and suggest superiority of toenail over hair analyses for these purposes. Our results also suggest there are changes in maternal and infant Mn biomarker levels that occur during the first year of the infant’s life, and there may be a need to identify specific time windows for sample collection in order to obtain meaningful data on Mn exposure. For instance, assessment of *in utero* exposure may need to rely on the analysis of toenail samples collected shortly after birth, while evaluation of Mn exposure over the first year of life may need to be based on toenail samples collected not earlier than 12 months after birth. Further research should focus on characterization of the impact of sample timing, maternal exposures, gestational age at delivery, and infants’ sex and Mn intake through drinking water and diet, on infant toenail Mn levels. Such knowledge is necessary for better understanding of how toenail Mn data can be used to accurately assess *in utero* and early childhood Mn exposures and link these exposures to health outcomes.

## Materials and Methods

### Human subjects

Study subject recruitment and sample collection were approved by the University of Minnesota (UMN) Institutional Review Board (IRB #1401M46943 and #1401M47181). All research was performed in accordance with relevant guidelines and regulations, adhering to the tenets of the Declaration of Helsinki. Informed consent was obtained from all mothers including consent to collect hair and toenail samples from their infants. Recruitment was carried out in two cities in Minnesota (U.S.) between July 2014 and March 2015. Both cities had high levels of naturally occurring Mn in ground water, but one city did not filter their municipal water supply for Mn, whereas the other city did and reported negligible levels of Mn in their drinking water supply. Additionally, the cities had comparable population demographics and birth rates. Infants who were born full-term (>36 weeks of gestation) from pregnancies void of fetal neurologic health risk factors, and mothers who resided in the selected cities during the entire pregnancy and through the time of developmental testing were enrolled in the study as infant-mother pairs. Recruitment occurred when infants were 7 months old (Scheme 1). Both toenail and hair samples were collected from infants and their mothers. Water samples were collected in a clean bottle from each mother’s residence, including the water used to reconstitute formula, if applicable. For a subset of these infant-mother pairs, samples were collected at 2 additional time periods relevant to *in utero* and early childhood exposure: prenatal toenail, hair, and venous blood samples from the mother during the third trimester of pregnancy, and a sample of cord blood at the time of birth. In addition, a single infant-mother pair with a history of high Mn exposure at early stages of pregnancy participated in a longitudinal monitoring of Mn biomarkers. The mother provided multiple biological samples at 6 different time points, from the third trimester of pregnancy until her child reached 9 months of age.

### Sample collection procedures

Mothers were provided sample collection kits with supplies and detailed instructions on how to collect their own and their infants’ toenail and hair samples. To eliminate potential variation in contamination of supplies with Mn, all mothers were given supplies from the same lot number. For toenail sample collection, stainless steel nail clippers were used to clip toenails from all ten toes, which was done over a clean paper towel to prevent external contamination. The toenails were then placed in a pre-labeled specimen bag and stored at room temperature. Hair samples were collected by using stainless steel scissors to cut about 50 strands of hair (or approximately 5 mm in diameter) from just above the nape of the neck or as near to the scalp as could be done safely. The end of the hair strand that had been close to the scalp was marked with a rubber band, and the samples were placed in a pre-labeled specimen bag and stored at room temperature. The collected toenail and hair samples were delivered to the UMN laboratory either by mail or by a public health nurse, who made a home visit.

Blood samples were collected into a metal-free trace Vacutainer® tube (BD, Franklin Lakes, NJ, USA) containing K_2_EDTA. Prenatal venous blood samples from the mothers were collected by the public health nurse during the prenatal visit. Cord blood was collected at the time of delivery. Blood samples were immediately brought to the UMN laboratory, processed, and stored in a −20 °C freezer.

### Analysis of Mn in biological samples

Washing and digestion procedures were carried out based on previously published procedures and with some modifications^27,33^. Toenails were briefly pre-washed by sonication in a 1% Triton™ X-100 solution (Millipore Sigma, St. Louis, MO, USA), followed by an additional wash in acetone and 3 rinses with deionized water. A similar procedure was used, except for the acetone wash, to pre-wash the hair samples. The whole length of hair samples collected from infants was used for analyses because of the very small mass of hair samples. For hair samples collected from mothers, only the first 2.5 centimeters from the scalp side were used to standardize the comparisons across women (there was inter-individual variation in the hair length) and because there was sufficient hair mass. Once cleaned, samples were dried overnight in a 60 °C drying oven, cooled, and weighed. The prepared toenail and hair samples, as well as blood samples (200 uL of whole blood), were then digested overnight at room temperature, using a 3:1 mixture of ultra-pure nitric acid and hydrochloric acid. Before analysis, each sample was diluted with deionized water to 6 mL. Due to the limited mass of toenails, all toenails from a participant were pooled together for analysis to maximize the ability to detect Mn.

Measurement of Mn in acid-digested samples was carried out by inductively coupled plasma-mass spectrometry (ELAN® DRC II; PerkinElmerSCIEX™, Norwalk, CT, USA) at the Minnesota Department of Health Public Health Laboratory. All samples were analyzed in triplicate, and the average of the 3 samples was used for data interpretation. Analytical accuracy was monitored by including UMN laboratory-prepared negative control (method blanks) and positive control (QC) samples with each batch of samples. Certified reference material that was available in our laboratory (Standard Reference Material Tomato Leaves NIST 1573a, National Institute of Standards and Technology, Gaithersburg, MD, USA) was used to prepare QC samples. All sample analytical values were blank-corrected to account for any analyte contamination that could be a result of reagents, instruments and/or analyst variability. Analysis of Mn in the drinking water samples was performed by the same method.

### Statistical analysis

Descriptive statistics (mean, standard deviation, minimum and maximum) were used to characterize Mn biomarker data between infants and their mothers. Pearson correlations (R) were calculated to assess the strength of the relationship between infant-mother biomarkers. A p-value of less than 0.05 was considered significant. Infant biomarkers and postnatal, maternal biomarkers were log-transformed using the natural logarithm to compute correlations. For the statistical analysis purposes, samples with non-detected Mn levels were assigned a value equal to half of the lowest amount of Mn measured in a sample. Data analysis was performed using Stata-IC statistical software (version 14; StataCorp LLC, College Station, TX, USA).

## Data Availability

The datasets generated during and/or analyzed during the current study are available from the corresponding author on reasonable request.
